# Supplementary material for: Different Microeukaryotic Trophic Groups Show Different Latitudinal Spatial Scale Dependences in Assembly Processes across the Continental Shelves of China
Source: Microorganisms. 2024 Jan 8;12(1):124. doi: 10.3390/microorganisms12010124 (PMC10821338; doi:10.3390/microorganisms12010124)
Supplement: Supplementary file 1 [file microorganisms-12-00124-s001.zip › Supplementary materials/Figure S2.pdf]

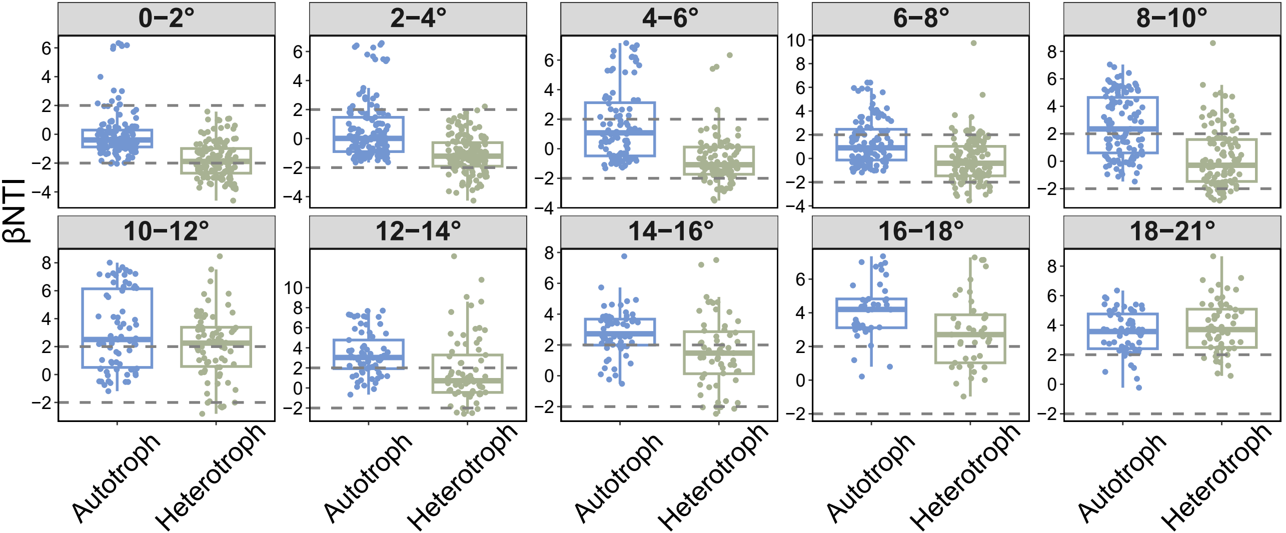

βNTI
0−2°
2−4°
4−6°
6−8°
8−10°
10−12°
12−14°
14−16°
16−18°
18−21°
Autotroph
Heterotroph
