# Supplementary figures and images for: Different Microeukaryotic Trophic Groups Show Different Latitudinal Spatial Scale Dependences in Assembly Processes across the Continental Shelves of China
Source: Microorganisms. 2024 Jan 8;12(1):124. doi: 10.3390/microorganisms12010124 (PMC10821338; doi:10.3390/microorganisms12010124)

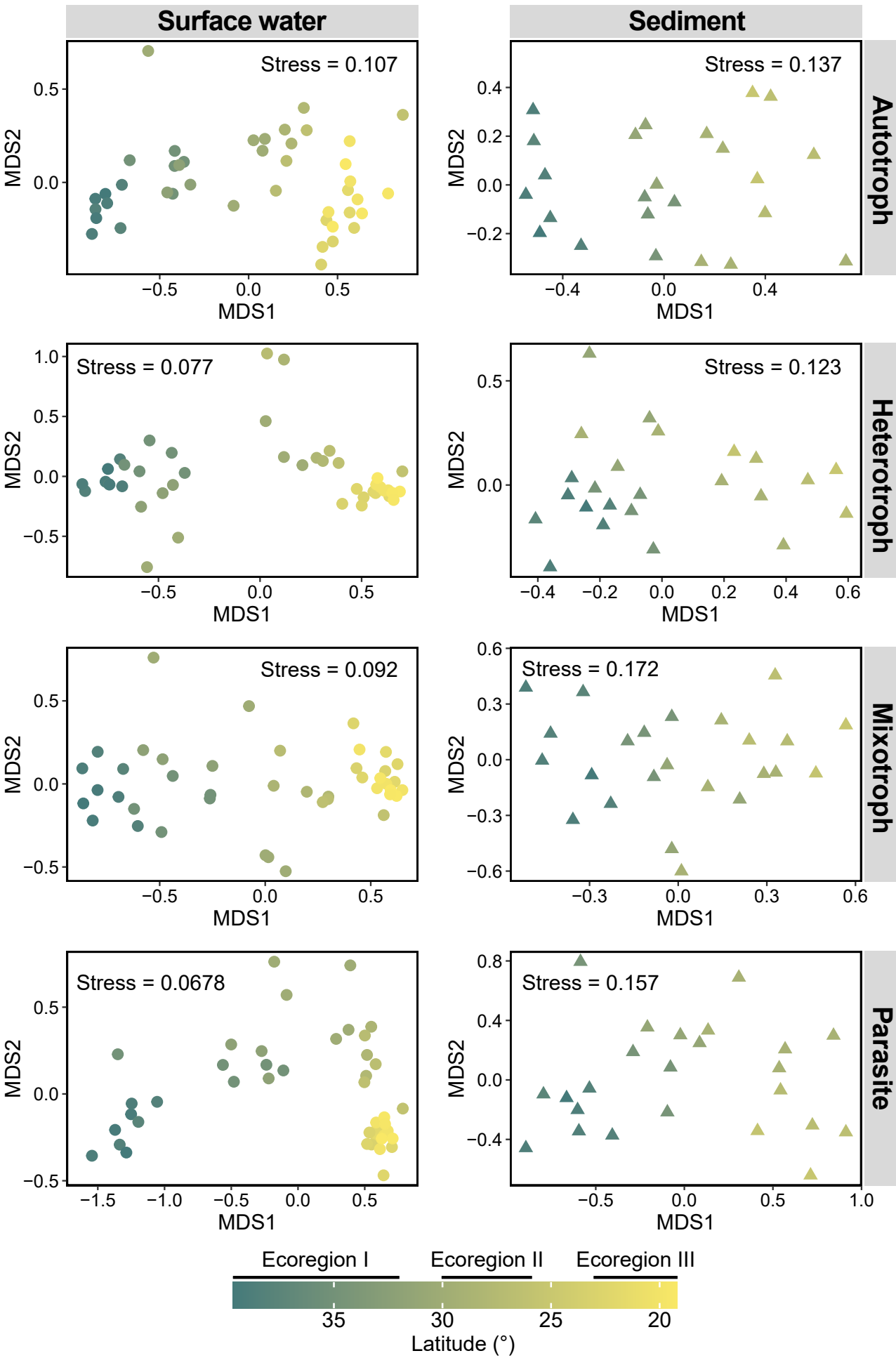

Supplement: Supplementary file 1 [file microorganisms-12-00124-s001.zip › Supplementary materials/Figure S1.pdf]
